# Improving the Description of Electronically Inelastic Scattering of Electrons by the Furan Molecule

**DOI:** 10.1021/acsphyschemau.5c00027

**Published:** 2025-07-29

**Authors:** Yan A. C. de Avó, Giseli M. Moreira, Romarly F. da Costa

**Affiliations:** † Centro de Ciências Naturais e Humanas, 74362 Universidade Federal do ABC, Santo André, São Paulo 09210-580 Brazil; ‡ Departamento de Física, Universidade Estadual do Centro-Oeste, Guarapuava, Paraná 85040-167 Brazil; § Departamento de Física, Universidade Federal do Paraná, Caixa Postal 19044, Curitiba, Paraná 81531-980 Brazil

**Keywords:** electron scattering, Schwinger multichannel method, multichannel coupling, furan, inelastic cross section

## Abstract

We present elastic and electronically inelastic cross-sections for low-energy electron scattering (up to 30 eV) by the gas-phase furan molecule. The calculated cross sections were obtained using the Schwinger multichannel method implemented with norm-conserving pseudopotentials. The influence of multichannel coupling effects was investigated by comparing four distinct scattering models, each employing a different channel coupling scheme. Our results for elastic and electronically inelastic scattering show excellent agreement with the available experimental data. For electronically inelastic collisions, despite the limited literature, the model with 197 channels demonstrates remarkable correspondence with experimental cross sections, highlighting the critical role of accurately accounting for multichannel coupling effects to obtain a reliable theoretical prediction for the corresponding cross-sections.

## Graphical abstract

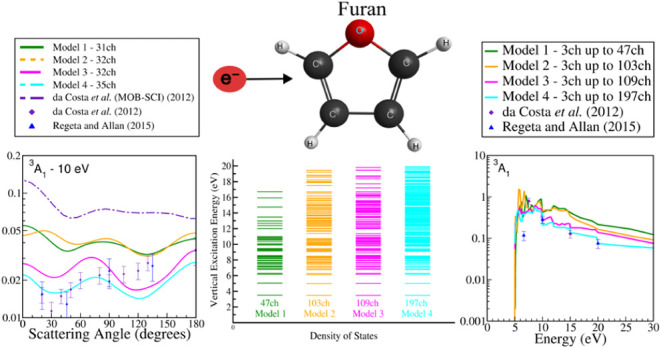

## 1 Introduction

Studies on electron-molecule collisions have a wide range of applications, including research on biofuel production,^1–3^ atmospheric discharges,^4,5^ plasmas for surface treatments,^6,7^ and radiotherapy.^8–10^ In cancer therapy, ionizing radiation is used as a genotoxic agent in therapeutic procedures. Upon interacting with matter, the energy carried by the ionizing radiation is rapidly thermalized as a result of a series of scattering processes with the biological tissue, potentially generating a range of secondary products. Among these secondary products, photons, ions, radicals, excited species, and a substantial amount of secondary electrons can be produced.^11–13^

Motivated by these findings, Boudaïffa and collaborators^14^ reported a study involving DNA damage resulting from collisions of low-energy secondary electrons (up to approximately 20 eV), which arise from the interaction of ionizing radiation with genetic material. According to these authors, most of the lesions occurring at impact energies below 15 eV are attributed to the temporary capture of the incident electron in some region of the DNA, followed by the cleavage of a specific bond and subsequent reactions involving fragmentation products. Following the dissemination of this work, theoretical and experimental groups have focused on investigating the scattering processes that involve electrons and the components of DNA and RNA, as well as simpler systems considered as prototypes of these molecules. Such investigations (see, for example, references 11–13 and 15–19 ) have demonstrated that DNA damage caused by secondary electrons can occur through direct mechanisms, such as ionization, vibrational excitation, electronic excitation, among others, as well as through indirect mechanisms via the formation of resonant states.

The study of electron scattering with basic DNA and RNA constituents, or with molecules considered as its prototypes, also has important applications in analyzing the biological effects of ionizing radiation, specifically in determining the precision of radiation doses used in cancer treatment. In this context, the development of more refined global models (Monte Carlo track structure simulations^20–24^ to describe radiolysis in cellular media) depends on a careful review and compilation of the databases that comprise the input parameters for such models. For each molecular component, this set of parameters includes total cross section (TCS), integral cross sections (ICSs), and differential cross sections (DCSs) for elastic scattering and all known inelastic scattering processes driven by electron impact, as well as energy-loss distributions.

Considering the overall scenario described above, we performed a theoretical study on low-energy electron collisions with the furan molecule. Furan is the simplest five-membered heterocyclic aromatic compound that contains an oxygen atom in its ring structure. We selected this molecule because it serves as a simpler yet analogous system to tetrahydrofuran, a sugar-like component of the DNA backbone.

The literature on the furan molecule is extensive. However, despite the considerable number of studies, significant gaps remain. We conducted a literature review, gathering data on the excited states and cross sections for electron scattering by furan molecules, and we outline key considerations regarding the state-of-the-art in these studies, as follows:

(1) Early investigations into the excited states of furan, notably by van Veen^25^ and Flicker et al.^26^ identified electronic excitation structures around 4.0 and 5.2 eV, with subsequent studies revealing higher-energy excited states ranging from 5.2 to 12 eV. Palmer et al.^27^ confirmed these findings through a combination of theoretical and experimental approaches, which showed to be in good alignment with calculated excitation energies, particularly when the basis sets include polarization functions.

(2) Studies that focus on the formation and characterization of resonant states during electron scattering have documented resonances at approximately 1.7 and 3.2 eV, although theoretical predictions tend to suggest slightly higher resonance energies compared to experimental observations. Notable contributions include those of Giuliani and Hubin-Franskin^28^ who used high-resolution electron energy loss spectroscopy (HREELS) to further elucidate the excitation spectrum.

(3) The cross sections for elastic electron scattering by furan have been extensively measured and calculated across energies from 0 eV to 10 keV. In particular, Bettega and Lima^29^ employed the Schwinger multichannel (SMC) method with pseudopotentials, which identified shape resonances at 2.1, 4.2, and 6 eV. Khakoo et al.^30^ corroborated these results, revealing resonances at 1.65 and 3.10 eV through their measurements. The consistency between theoretical and experimental cross sections is particularly strong for energies below 7 eV and above 50 eV, while discrepancies are observed between 7 and 50 eV, with theoretical values generally exceeding the experimental data.

(4) Comparatively, literature on electronically inelastic electron scattering remains limited. However, studies by da Costa et al.^31,32^ and Regeta and Allan^33^ have provided data on differential and integral cross sections for the lowest excited states of furan. Notably, da Costa et al. employed the SMC method, yielding results that were consistent with experimental measurements near the electronic excitation thresholds, although the calculated cross sections significantly exceeded the experimental values at higher energies, especially above 6 eV.

Based on the above, the present level of understanding on the subject is highly favorable to the investigations proposed in this work. In fact, while for elastic scattering the electron-molecule interaction process is well documented and provides a benchmark to assess the level of accuracy of the current scattering calculations, the situation regarding electronic excitation of molecules by electron impact is far from satisfactory. Notably, there is limited literature on electronically inelastic scattering and substantial discrepancies also exist between measured cross sections and theoretical predictions, especially at the impact energy of 10 eV and above this value. Although the results for the electronic excitation of furan by electron impact are limited, their presence in the literature will facilitate a more thorough evaluation of the results obtained in this work. Having these considerations in mind, we present DCSs and ICSs for both elastic and electronically inelastic scattering obtained by using the SMC method with norm-conserving pseudopotentials. Our primary focus is on electronically inelastic scattering, as previous work has demonstrated good agreement between calculated and experimental elastic cross sections. However, since the elastic channel always participates in the competition for the probability flux that defines the cross section as an energetically accessible state, ensuring that this scattering process is adequately described in the different models used in this study is a crucial step. To fully achieve this purpose and evaluate the influence of multichannel coupling effects on the magnitude of the cross sections, we carried out calculations using four distinct scattering models, each employing a different channel coupling scheme. A critical comparison of the cross sections obtained for both elastic and electronically inelastic scattering with the data available in the literature data will help to further assess the impact of polarization and multichannel coupling effects on the cross sections.

The remainder of this paper is organized as follows. In Section 2, we report on the theoretical method, and the computational procedures used to carry out the scattering calculations. We present and discuss our results in Section 3, and finally, in Section 4, we close the paper with a brief summary of the present findings.

## 2 Methods

### 2.1 Theory

Elastic and electronically inelastic calculations were performed using the SMC method^34,35^ implemented with the norm-conserving pseudopotentials of Bachelet, Hamann, and Schlüter (BHS).^36^ While detailed descriptions of the method are available in the literature,^34,37,38^ we will outline only the key aspects relevant to the present calculations.

The SMC method provides a variational approximation for the scattering amplitude in electron-positron collisions with molecules. However, in this work, the method is specifically applied to electron scattering. In this case, the resulting expression for the scattering amplitude is given by

1 $$f\left({\mathrm{k⃗}}_{f},{\mathrm{k⃗}}_{i}\right)=-\frac{1}{2\pi }\underset{m,n}{\sum }\left\langle S_{{\mathrm{k⃗}}_{f}}\left|V\right|\chi_{m}\right\rangle (d^{-1}{)}_{mn}\left\langle \chi_{n}\left|V\right|S_{{\mathrm{k⃗}}_{i}}\right\rangle$$

where $\left|S_{{\mathrm{k⃗}}_{i,f}}\right\rangle$ is a solution of the unperturbed Hamiltonian *H*_0_, written as the product of the target state and a plane wave with momentum ${\mathrm{k⃗}}_{i,f}$ ; *V* is the interaction potential between the incident electron and the target; the trial vectors |χ_*m*_⟩ are (*N* + 1) electron configuration state functions (CSFs), built from the product of the target electronic states with the single-particle functions and *d*_*mn*_ are the matrix elements of the *A* ^(+)^ operator in the {χ_*m*_} basis given by

2 $$d_{mn}=\left\langle \chi_{m}\left|A^{\left(+\right)}\right|\chi_{n}\right\rangle$$

with the operator *A* ^(+)^ written as

3 $$A^{\left(+\right)}=\frac{1}{2}\left(PV+VP\right)-VG_{P}^{\left(+\right)}V+\frac{1}{N+1}\left[Ĥ-\frac{N+1}{2}\left(ĤP+PĤ\right)\right]$$

In the expression above, *P* is the projection operator onto the open-channel space, $G_{P}^{\left(+\right)}$ is the free-particle Green function projected onto the *P*-space and Ĥ=E−H is the collision energy minus the Hamiltonian of the electron-target system, with *H* = *H*_0_ − V.

The ICS for the transition process Φ_*n*_ → Φ_*n*’_ (where, as will be defined below, Φ_*n*_ denotes an electronic state of the target) can be obtained from the scattering amplitude given in eq 1 such that

4 $$\sigma_{n\to n^{\prime }}\left(E\right)=\frac{1}{4\pi }\frac{k_{f}}{k_{i}}\int d{k̂}_{i}\int d{k̂}_{f}|f\left({k⃗}_{f}^{\prime },{k⃗}_{i}\right){|}^{2}$$

where the magnitude of the final wave vector is given by $k_{f}^{2}=k_{i}^{2}-2\left(\varepsilon_{n}-\varepsilon_{n^{\prime }}\right)$ , with *ε*_*n*_ being the energy of the *n*-th electronic target state. The integration in ${\mathrm{k̂}}_{f}$ accounts for the scattering into all the possible directions, while the term ${\left(4\pi \right)}^{-1}\int d{\mathrm{k̂}}_{i}$ averages the random molecular orientations in the target gas. By integrating over all incoming and outgoing directions, the ICS becomes rotationally invariant. This invariance ensures that the ICS is the same regardless of whether it is determined in the body-fixed frame or the laboratory-fixed frame.

In contrast, to allow for a direct comparison with the measurements, the DCSs were obtained by expanding the scattering amplitude, hereafter referred to as *f* ^*BF*^(k⃗_*f*,_k⃗_*i*_) in partial waves (see, for instance, ref ^39^):

5 $$f^{BF}\left({\mathrm{k⃗}}_{f},{\mathrm{k⃗}}_{i}\right)=\overset{l_{max}}{\underset{l,m}{\sum }}F_{l,m}^{BF}\left(k_{f},{\mathrm{k⃗}}_{i}\right)Y_{l}^{m}\left({\mathrm{k̂}}_{f}\right)$$

with

6 $$F_{l,m}^{BF}\left(k_{f},{\mathrm{k⃗}}_{i}\right)=\int d{\mathrm{k̂}}_{f}{Y_{l}^{m}}^{*}\left({\mathrm{k̂}}_{f}\right)f^{BF}\left({\mathrm{k⃗}}_{f},{\mathrm{k⃗}}_{i}\right)$$

and where the superscript *BF* indicates that in our calculations the scattering amplitude is determined in the body reference frame so as to take advantage of the symmetry properties of the molecular target. Then, we performed the appropriate transformations to express the scattering amplitude in terms of the laboratory frame (*LF*) angles:

7 $$f^{LF}\left({k⃗}_{f}^{\prime },{k⃗}_{i}\right)=\overset{l_{max}}{\underset{l,m,\mu }{\sum }}F_{l,m}^{BF}\left(k_{f},{k⃗}_{i}\right)Y_{l}^{\mu }\left({k̂}_{f}^{\prime }\right)D_{m,\mu }^{l}\left(0,\beta ,\alpha \right)$$

where D⃗ stands for the Wigner rotation matrices whose argument consists of the Euler angles relating the two reference frames, ${k⃗}_{i}=\left(\beta ,\alpha \right)$ and ${k̂}_{f}^{\prime }=\left(\theta^{\prime },\phi^{\prime }\right)$ are the scattering angles in the laboratory frame. The random orientation of the target is accounted for by averaging over the incident (body frame) angles also denoted by ${\mathrm{k̂}}_{i}$ , that is,

$$\frac{d\sigma }{d\Omega }\left(\theta^{\prime },\phi^{\prime },k_{f},k_{i}\right)=\frac{1}{4\pi }\frac{k_{f}}{k_{i}}\int d{\mathrm{k̂}}_{i}|f^{LF}\left({{k⃗}_{f}}^{\prime },{k⃗}_{i}\right){|}^{2}$$ 8

For a transition of interest, the DCSs are obtained by averaging the above expression over the azimuthal angle ϕ' and performing the appropriate average over the initial and sum over the final spin states.

### 2.2 Computational Details

The ground state geometry of furan, obtained from the NIST database ^40^ was optimized in the *C*_2v_ point group by using the TZV++(3d,1p) basis set and the second order Møller-Plesset (MP2) perturbation theory as implemented in the GAMESS^41^ computational package. The resulting lowest energy structure provided geometric parameters in very good agreement with the calculated values reported by Gavrilov et al.^42^ and Jagau et al. ^43^ with discrepancies being less than 0.005 Å for the bond lengths and 0.3^◦^ for the angles. Having obtained the optimal geometry, the electronic ground state of the target was described within the Hartree–Fock level of approximation. In Figure 1 we present the geometrical structure of the target, visualized using the MacMolPlt^44^ interface. The single particle basis set employed in our calculations was generated according to Bettega et al.^37^ for oxygen and carbon atoms (Table 1) and according to Dunning^45^ for hydrogen atoms (Table 2). To provide a better description of the excited states of the furan molecule, we used the improved virtual orbitals (IVOs)^46^ obtained from a cationic Fock operator with charge +1, to represent the particle and scattering orbitals.

**Figure 1:**
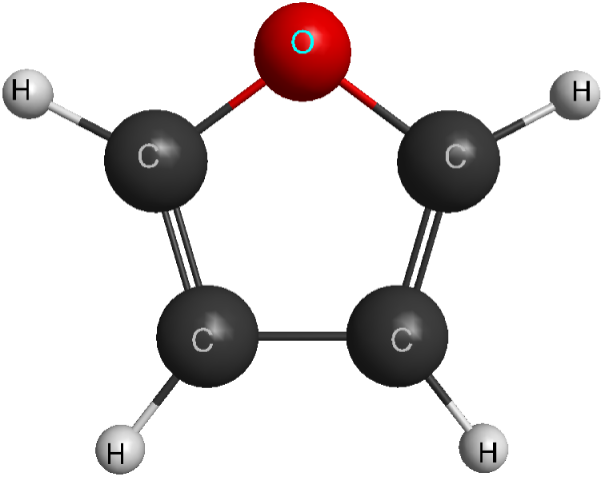
Ball-and-stick model of the furan molecule. Generated with MacMolPlt.^44^

**Table 1:** Exponents of the Uncontracted Cartesian Gaussian (CG) Functions Used for Oxygen and Carbon Atoms

Type	Oxygen	Carbon
*s*	16.058780	12.496280
*s*	5.920242	2.470286
*s*	1.034907	0.614028
*s*	0.316843	0.184028
*s*	0.065203	0.039982
*p*	10.141270	5.228869
*p*	2.783023	1.592058
*p*	0.841010	0.568612
*p*	0.232940	0.210326
*p*	0.052211	0.072250
*d*	0.756793	0.603592
*d*	0.180759	0.156753

**Table 2:** Exponents and Coefficients of the Cartesian Gaussian (CG) Functions Used for Hydrogen Atoms

Type	Exponents	Coefficients
*s*	13.361500	0.130844
*s*	2.013300	0.921539
*s*	0.453800	1.000000
*s*	0.123300	1.000000
*p*	0.750000	1.000000

In this study, the scattering calculations were carried out within the minimal orbital basis for single configuration interactions (MOB-SCI) strategy.^47^ In this approach, the target states included in the open channel space are represented using a minimal basis of individually excited spin-adapted Slater determinants, carefully chosen to closely replicate the energy of the first electronically excited states. To apply this strategy, the energy spectrum of the electronically excited states is first obtained according to a complete single configuration interaction (FSCI) calculation, which considers all single excitations of the electrons in the molecule and uses the IVOs to represent the unoccupied molecular orbitals of the target. Then, the excited states chosen to be included in the scattering calculations are described using a small number of hole-particle pairs that contribute the most to each excited state, where the hole corresponds to the electron initially in an occupied orbital that is promoted to an unoccupied (particle) orbital. Although reduced in comparison to the total number of pairs appearing in the FSCI representation of the selected excited states, the hole-particle pairs used in the MOB-SCI strategy match at least 90% of the corresponding FSCI energy value, ensuring consistency between methods. The vertical excitation energies for the first 15 excited states of the furan molecule obtained within the scope of the FSCI and MOB-SCI techniques are listed and, whenever possible, compared to available theoretical and experimental data from the literature in Table S1. For a given set of *N* hole-particle orbitals, the total number of channels considered is (2*N* + 1).

To explore the influence of the multichannel coupling effects upon the scattering process, we designed four models with increasing complexity. These models differed in (i) the number of excited states from the FSCI calculation that were selected to be described in the MOB-SCI approximation; (ii) the sets of hole-particle orbitals used to describe those states; and (iii) the total number of channels and configuration state functions (CSFs) resulting from this approximation for each symmetry. The progression across the models involved a systematic increase in the number of excited states included in the MOB-SCI scattering calculations, with the simplest model containing only a few excited states and the most robust model quadrupling the number of excited states and orbital pairs. This increase was intended to assess whether and how the additional number of hole-particle pairs used in the description of excited states included in the coupled channel space and of channels included in the scattering calculation in going from the simplest to the more complex models significantly improves the agreement with experimental data. In the following, we will provide details on the different models employed.

Using the FSCI strategy, we obtained a total of 2223 excited states in the furan spectrum. These states were arranged in ascending order of energy. Models 1 and 2 were designed to describe the electronically excited states obtained using the FSCI strategy with energies below the first ionization threshold of the furan molecule, at approximately 8.9 eV.^40^ This selection yielded 22 excited states in total. From these states, we identified the most significant contributions by selecting 23 hole-particle orbital pairs for model 1 and 51 hole-particle orbital pairs for model 2. In order to increase the number of excited states to be described, we constructed models 3 and 4. In model 3, we selected the five lowest-energy excited states for each irreducible representation (according to the *C*_2*v*_ point group) and spin coupling, resulting in a total of 40 excited states. Similarly, in model 4, we included the ten low-lying excited states for each irreducible representation and spin coupling, increasing the total to 80 excited states. For these expanded models, the corresponding hole-particle orbital pairs were selected such that we used 54 orbital pairs for model 3 and 98 orbital pairs for model 4. These choices allowed for a broader representation of the molecule’s excitation spectrum and its interaction with incident electrons.

A consistent energy cutoff of 20 eV was applied across all models to ensure that all electronically excited states remain energetically accessible to incident electrons within the range of energies considered in our work. This cutoff ensures that all relevant scattering channels remain open at or below 20 eV, while excluding pseudostates originating from higher-energy excitations that are not pertinent to low-energy electron scattering processes. A summary of the relevant information about models 1 to 4 (such as number of states, number of coupled channels, and number of CSFs per symmetry) is provided in Table 3. More details regarding these models, as well as a schematic representation of the density of states obtained according to each of them, can be found in Table S2 and Figure S1 respectively.

**Table 3:** Number of FSCI Excited States Being Described, Channels and Configuration State Functions per Symmetry for Each of the Multichannel Coupling Models Considered in This Work

			CSFs			
Calculations	FSCI States	Channels	*A*_1_	*A*_2_	*B*_1_	*B*_2_
Model 1	22	47	978	1063	995	1068
Model 2	22	103	2047	2366	2083	2396
Model 3	40	109	2747	1928	2785	1945
Model 4	80	197	4728	3742	4724	3735

The scattering calculations were carried out at a level of approximation in which the (*N* + 1)-electron basis set is given by

9 $$\left|\chi_{rm}\right\rangle =A_{N+1}\left|\Phi_{r}\right\rangle ⊗\left|\phi_{m}\right\rangle$$

where *r* = 0, ..., *n* and *n* is the number of open channels. $A_{N+1}$ is the antisymmetrizer operator; |Φ_*r*_⟩ are *N*-electron Slater states obtained by carrying out single excitations of the target electrons from the occupied (hole) orbitals to a set of unoccupied (particle) orbitals and |*ϕ*_*m*_⟩ is a single-particle orbital used to represent the scattering orbitals.

In the present calculations, the polarization effect is accounted for through virtual excitations of the target. As the number of channels used in each of the models we have used increases, the corresponding number of configurations also expands and, consequently, a more accurate description of the polarization effects is expected. Moreover, we anticipate that models incorporating a greater number of excited states and hole-particle orbital pairs will result in improved agreement with the experimental cross sections available in the literature, compared both to previous calculations employing the SMC method and to the simpler models here proposed.

## 3 Results and Discussion

### 3.1 Elastic Scattering

In Figure 2 we present the DCSs obtained with the SMCPP method according to models 1 to 4 at the impact energies of 10 and 30 eV. For comparison purposes, we also included in this figure the DCS data available in the literature. In the four models considered in this work, the cross sections at 10 eV (Figure 2a) exhibit a smaller magnitude for scattering angles above approximately 60^◦^ compared to the theoretical results of Bettega and Lima,^29^ Khakoo et al.,^30^ and da Costa et al.^31^ It is important to note that the results reported by Bettega and Lima ^29^ as well as by Khakoo et al. ^30^ were obtained without accounting for multichannel coupling effects. In contrast, da Costa et al.^31^ determined the cross sections within a multichannel coupling approximation, considering the coupling of up to nine channels. Present work extends this approach by incorporating a more extensive number of coupled channels. In general, our calculations show excellent agreement with the measurements of Khakoo et al.^30^ and Regeta and Allan^33^ with at least one of the curves matching nearly all the experimental results throughout the entire angular range. At the impact energy of 30 eV (Figure 2b), the shape and magnitude of the cross sections across all models closely align with the results reported by da Costa et al.^31^ Although the general trends are consistent among these models, the most comprehensive (197 channels) model exhibits a slightly lower magnitude compared to the others and demonstrates the best agreement with the measurements of Khakoo et al.^30^ with an almost identical shape and magnitude throughout the angular range.

**Figure 2:**
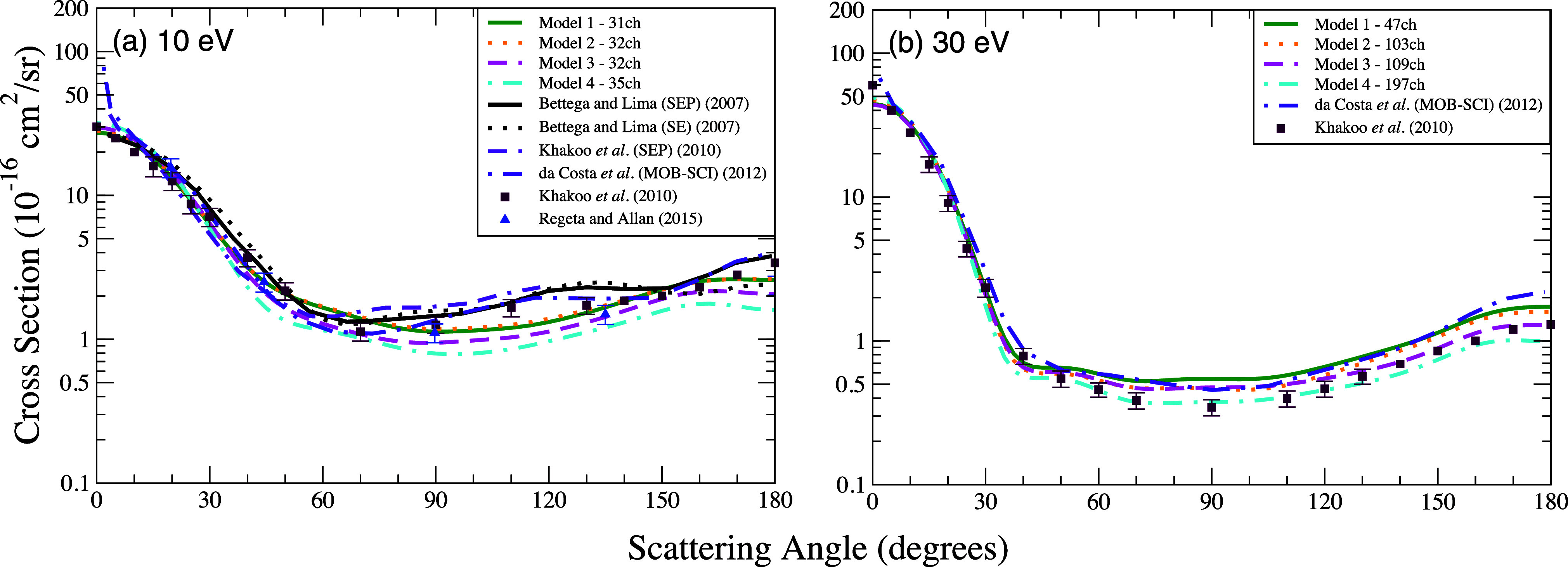
Differential cross section for elastic scattering of electrons by furan at the energies of (a) 10 eV and (b) 30 eV. Calculations were performed according to the following models for inclusion of the multichannel coupling effects: 47 channels (solid green line), 103 channels (dotted orange line), 109 channels (dashed magenta line), 197 channels (dash-dotted cyan line). Results from the literature were obtained experimentally by Khakoo et al.^30^ (dark brown square) and by Regeta and Allan^33^ (blue triangle) and theoretically using the SMC method by Bettega and Lima^29^ (dashed black line), by Khakoo et al.^30^ (dash–dash-dot violet line) and by da Costa et al.^31^ (dash-dotted indigo line).

In Figure 3, we present the ICS for elastic scattering over impact energies ranging from 1 to 30 eV. The influence of the multichannel coupling effect on the cross section begins to be evident from the threshold of the first electronically excited state, at approximately 3.5 eV. This effect becomes particularly significant beyond 5 eV, when multiple electronically excited states are energetically accessible to the electron-molecule system. For impact energies above 5 eV, the cross sections for the four models exhibit reduced magnitudes compared to those reported by Bettega and Lima ^29^ Khakoo et al.^30^ and Maljković et al.^48^ This difference is largely attributed to the inclusion of the multichannel coupling in our models, an effect that leads to a reduction in the magnitude of the ICSs due to the competition among a given number of states for the flux that defines the cross section, as previously discussed. Overall, these models demonstrate good agreement with the measured cross sections across most of the energy range, with at least one model consistently falling within the experimental uncertainty bounds.

**Figure 3:**
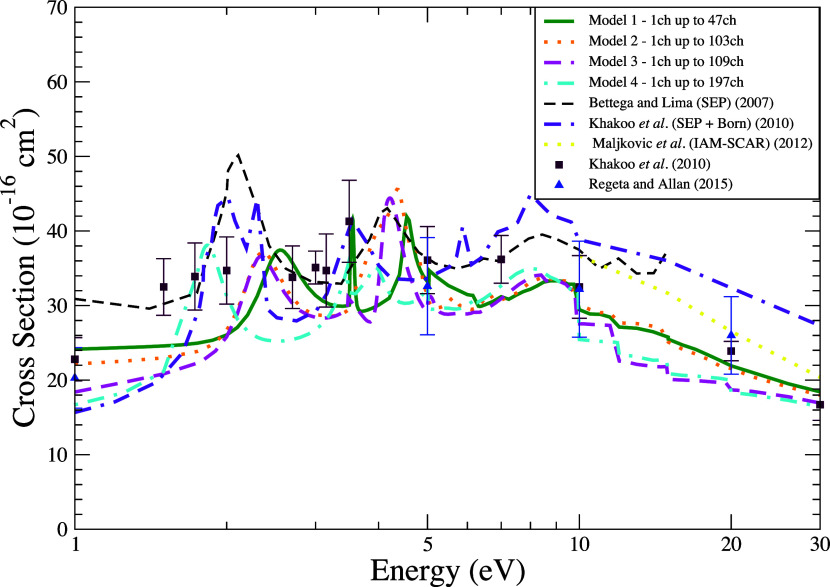
Integral cross section for elastic scattering of electrons with energies ranging from 1 to 30 eV by the furan molecule. Calculations were performed according to the following models for inclusion of the multichannel coupling effects: 47 channels (solid green line), 103 channels (dotted orange line), 109 channels (dashed magenta line), 197 channels (dash-dotted cyan line). Results from the literature were obtained experimentally by Khakoo et al.^30^ (dark brown square) and by Regeta and Allan^33^ (blue triangle) and theoretically using the SMC method by Bettega and Lima^29^ (dashed black line), by Khakoo et al.^30^ (dash–dash-dot violet line), and by da Costa et al.^31^ (dash-dotted indigo line) and using the IAM-SCAR method by Maljkovic et al.^48^ (dotted yellow line).

We also observe the presence of pronounced structures in our ICS results for energies below 10 eV. Typically, the peaks associated with resonances shift to lower energies as the treatment of polarization effects is improved, progressing from model 1 (up to 47 channels) to model 4 (up to 197 channels). It is important to note that nonphysical (spurious) structures may also exhibit shifts in their positions as a result of changes in the level of polarization. The positions of the pronounced structures observed in our ICSs as well as the assignments reported in previous works are listed in Table 4. The peaks located at around 1.96–2.62 eV and 3.69–4.25 eV exhibit the usual behavior for structures associated with resonances, as they tend to shift to lower energies with an improved description of polarization effects in our calculations. Considering these results and the proximity of these peaks to the resonances measured at approximately 1.7 and 3.2 eV, we attribute them to resonances. Further analysis in terms of the eigenvalues, obtained through diagonalization of the scattering Hamiltonian of the *H*_*N*+1_ electrons in the CSF space of the resonant symmetries, confirmed these assignments. Such diagonalization allows us to identify the resonant states of the *N*+1 electron system, as well as, constructed the single-particle orbitals from the eigenstates of *H*_*N*+1_. Although not shown here, the resonances that appear in the ICSs originate from the *B*_1_ and *A*_2_ symmetries, according to the *C*_2*v*_ point group, and these assignments are in agreement with previous works available in the literature.^29,30^

**Table 4:** Resonance Positions (In eV) Obtained in the Cross Sections from the Four Multichannel Coupling Models Considered in This Work^a^ ^b^ ^c^

	Multichannel Coupling Models				Experiment		Theory	
Resonances	47 ch	103 ch	109 ch	197 ch	^49^	^30^	^30^	^29^
$\pi_{1}^{*}$ (*ε*)	2.62	2.47	2.43	1.96	1.73	1.65	1.95	2.10
$\pi_{1}^{*}$ (ICS)	2.55	2.35	2.34	1.83				
$\pi_{2}^{*}$ (*ε*)	4.25	4.12	4.07	3.69	3.15	3.10	3.56	4.20
$\pi_{2}^{*}$ (ICS)	4.10	4.00	4.16	3.47				

a The first resonance belongs to the *B*_1_ symmetry, while the second belongs to the *A*_2_ symmetry.

b We also show the eigenvalue (*ε*) associated with the diagonalization of the scattering Hamiltonian (*H*_*N*+1_) in the CSF space.

c For comparison, we include the values measured by Modelli and Burrow^49^ and Khakoo et al.,^30^ as well as those calculated by Khakoo et al.^30^ and Bettega and Lima.^29^

Although the identification and characterization of resonant states are not the focus of our work, it is noteworthy that the positions for the center of the resonances appearing in the ICS obtained according to model 4 exhibit the best agreement with the experimental assignments among the four models. In particular, the value of 1.96 eV obtained for the position of the first resonance in this model is in very good agreement with the experimental data reported at, approximately 1.7 eV. This finding provides an indication that, among the models considered in this work, model 4 is the one that allows the best description of polarization effects for description of elastic electron scattering from furan in the low-energy regime. As mentioned before, by ensuring an adequate description of the elastic process, we made sure that important aspects of the scattering dynamics (such as the construction of the CSF space and the inclusion of polarization effects for determining the resonant structures) were incorporated in a balanced way. Therefore, based on these results, the evaluation of the influence of multichannel coupling effects on the electronic excitation processes can be conducted with confidence.

### 3.2 Electronically Inelastic Scattering

We present in Figure 4 the DCSs that involve the transitions from the ground state to the first excited state ^3^ *B*_2_ of the furan molecule for the energies of 5, 6, 10, and 20 eV. At 5 eV (Figure 4a), the results show that the cross sections from all the calculations, including that one obtained by da Costa et al.^31^ exhibit considerable differences in magnitude, which are partially attributed to the presence of pronounced structures in the integral cross sections near this energy (Figure 6). For the impact energies of 6 eV (Figure 4b) and 10 eV (Figure 4c), the models utilizing the same strategies for selecting the excited states of the FSCI approximation (models 1 and 2 as well as models 3 and 4) exhibit very similar DCSs. Although not previously mentioned, the similarity in the magnitude of the cross sections for models 1 and 2, as well as for models 3 and 4, is also evident in the elastic scattering results shown in Figures 2 and 3. In contraposition, for the impact energy of 20 V (Figure 4d) the calculations with a similar number of channels (models 2 and 3) resulted in similar cross sections.

**Figure 4:**
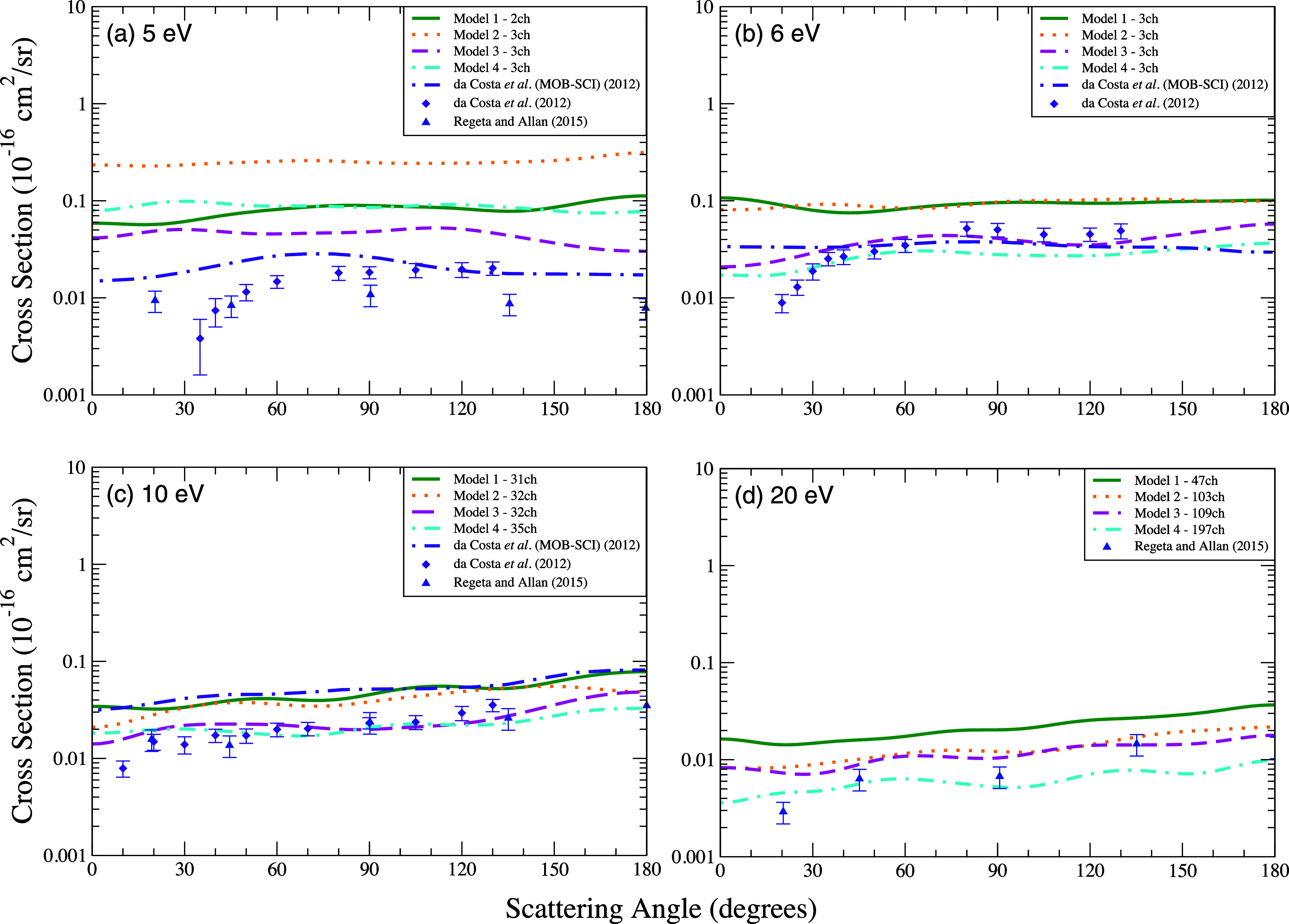
Differential cross section for electronically inelastic scattering of electrons by the furan molecule, specifically for the first electronically excited state (^3^ *B*_2_) of the molecule, at the energies of (a) 5 eV, (b) 6 eV, (c) 10 eV, and (d) 20 eV. Calculations were performed according to the following models for inclusion of the multichannel coupling effects: 47 channels (solid green line), 103 channels (dotted orange line), 109 channels (dashed magenta line), 197 channels (dash-dotted cyan line). Results from the literature were obtained theoretically by da Costa et al.^31^ using the SMC method (dash-dotted indigo line) and experimentally by da Costa et al.^31^ (indigo diamond) and by Regeta and Allan^33^ (blue triangle).

Compared to the experimental data reported by da Costa et al.^31^ and Regeta and Allan ^33^ the results obtained using models 3 and 4, show an excellent accord (both in shape and magnitude), mainly at 6, 10 and 20 eV. Although the calculations by da Costa et al.^31^ are closer to the experimental data at 5 eV, the agreement between measured and calculated cross sections is clearly improved for the energies of 6 and 10 eV when the current DCSs are compared to those obtained in previous work from ref ^31^.

The DCSs concerning the excitation from ground state to the second excited state (^3^ *A*_1_) of the furan molecule are displayed in Figure 5 at the selected energies of 10 and 20 eV. These results show general trends similar to that observed in the case of the first excited state, i.e., at 10 eV (Figure 5a) the DCSs for models 1 and 2 have approximately the same shape and magnitude, as does the comparison between models 3 and 4; at 20 eV (Figure 5b) the DCSs show similar behavior in terms of shape. Specifically, the DCSs for model 4 display an excellent agreement in terms of magnitude and shape with the experimental data. Although not shown here, the results for the DCSs at energy 7.5 eV (especially those corresponding to models 3 and 4) also show excellent agreement with the experimental data reported by da Costa et al.^31^

**Figure 5:**
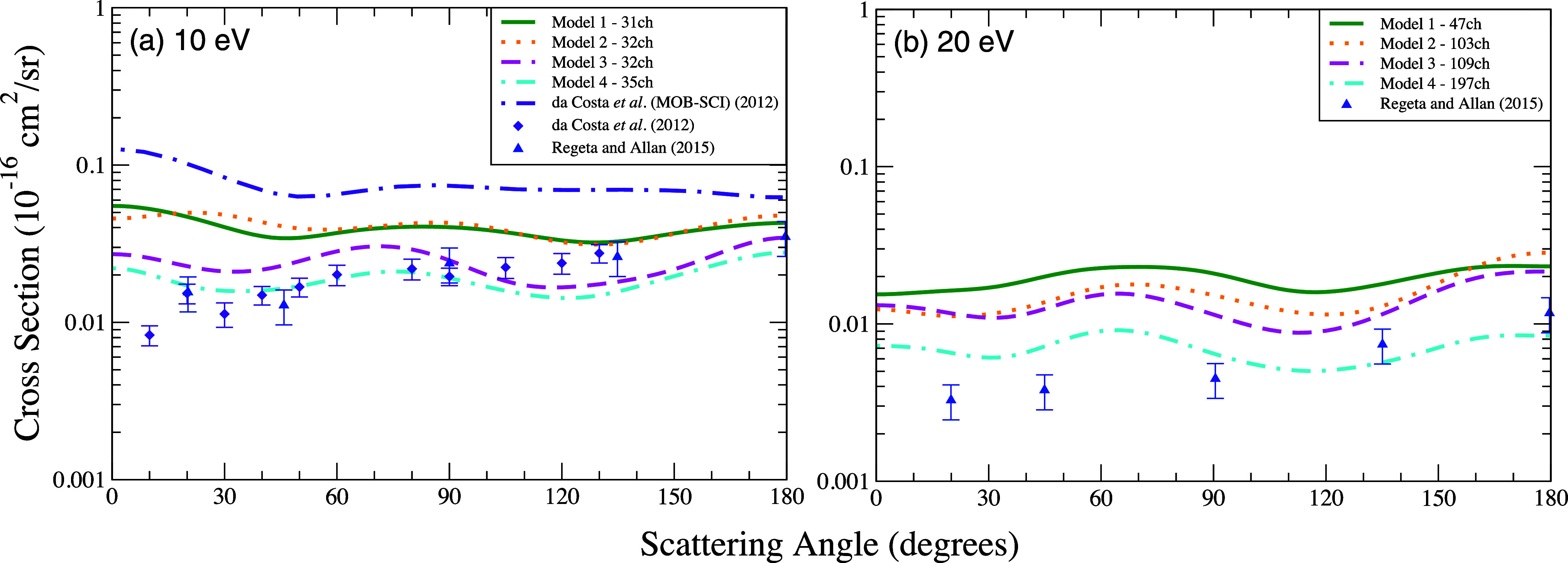
Differential cross section for electronically inelastic scattering of electrons by the furan molecule, specifically for the second electronically excited state (^3^ *A*_1_) of the molecule, at the energies of (a) 10 eV and (b) 20 eV. Calculations were performed according to the following models for inclusion of the multichannel coupling effects: 47 channels (solid green line), 103 channels (dotted orange line), 109 channels (dashed magenta line), 197 channels (dash-dotted cyan line). Results from the literature were obtained theoretically by da Costa et al.^31^ using the SMC method (dash-dotted indigo line) and experimentally by da Costa et al.^31^ (indigo diamond) and by Regeta and Allan^33^ (blue triangle).

Figures 6 and 7 show the ICSs results for the excitation into the first and second excited states, respectively, of furan over impact energies up to 30 eV. As already observed in the case of DCSs, the ICSs obtained by models 1 and 2 exhibit a similar shape and magnitude for energies up to 20 eV. Beyond this range, from 20 to 30 eV, the results obtained according to the intermediary models (models 2 and 3) display greater similarity to each other. Compared with the data available in the literature, for impact energies near the threshold of the first two excited states (≈3.5 and 5.0 eV, respectively), the magnitude of present ICS is higher than the experimental results reported by da Costa et al.^31^ and Regeta and Allan.^33^ Once again, this difference can be attributed to the presence of several pronounced peaks in our ICSs within this energy range. The assignment for the structures that appear in the ICSs presented in Figures 6 and 7 was not provided because, to date, we do not have a well-established procedure to define whether these peaks correspond to physical (resonances) or spurious structures. We also note that the results from model 4, which is our most robust model, show an excellent agreement with the experimental data as of 6 eV, with the cross sections for this model remaining within the experimental uncertainty range across nearly all measurements. Finally, it is also worth noting that model 4 provides an ICS with magnitude considerably smaller than the theoretical results previously reported by da Costa et al.^31^

**Figure 6:**
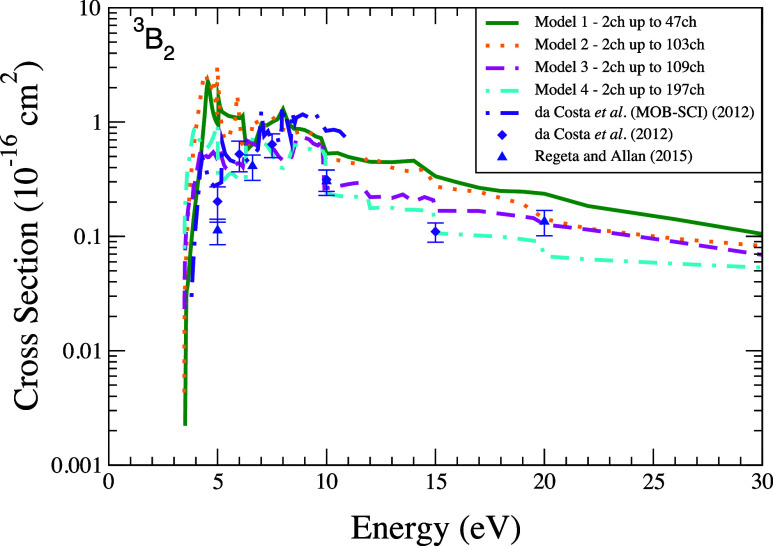
Integral cross section for electronically inelastic scattering of electrons by the furan molecule, specifically for the first electronically excited state of the molecule (^3^ *B*_2_). Calculations were performed according to the following models for inclusion of the multichannel coupling effects: 47 channels (solid green line), 103 channels (dotted orange line), 109 channels (dashed magenta line), and 197 channels (dash-dotted cyan line). Results from the literature were obtained theoretically by da Costa et al.^31^ using the SMC method (dash-dotted indigo line) and experimentally by da Costa et al.^31^ (indigo diamond) and by Regeta and Allan^33^ (blue triangle).

**Figure 7:**
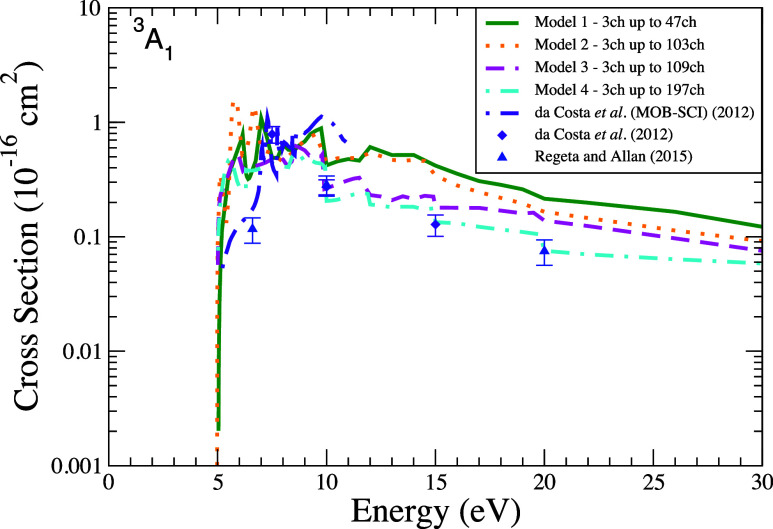
Integral cross section for electronically inelastic scattering of electrons by the furan molecule, specifically for the second electronically excited state of the molecule (^3^ *A*_1_). Calculations were performed according to the following models for inclusion of the multichannel coupling effects: 47 channels (solid green line), 103 channels (dotted orange line), 109 channels (dashed magenta line), 197 channels (dash-dotted cyan line). Results from the literature were obtained theoretically by da Costa et al.^31^ using the SMC method (dash-dotted indigo line) and experimentally by da Costa et al.^31^ (indigo diamond) and by Regeta and Allan^33^ (blue triangle).

Comparing the cross sections obtained for the four multichannel coupling models for both excited states of the furan molecule, we observe that differences in the strategy for selecting excited states using the MOB-SCI approximation, as well as the number of channels included in each model, can significantly affect the cross sections.

## 4 Conclusions

We presented cross sections for elastic and electronically inelastic electron scattering by the furan molecule calculated by using the SMC method implemented with the BHS pseudopotentials. The results were obtained according to the MOB-SCI strategy, considering four different models to describe the multichannel coupling effects. The number of open channels considered across the models varied from 1 to 197, with the maximum number of open channels depending on the chosen model.

Regarding the cross section data for the elastic electron scattering by the furan molecule, models 3 and 4 generally show excellent agreement with the experimental measurements reported by Khakoo et al.^30^ and Regeta and Allan.^33^ For impact energies of up to 10 eV, the multichannel coupling models with the best description of polarization effects (models 3 and 4) show greater consistency with the available literature, although the resonance positions in the calculations are still slightly higher by an amount of 0.10–0.18 eV in case of the $\pi_{1}^{*}$ and of 0.32–0.37 eV in case of the $\pi_{2}^{*}$ resonance than the reported experimental data. For energies above 10 eV, the similarity of all multichannel coupling models to the measurements carried out by Khakoo et al. and Regeta and Allan is remarkably good, with at least one of the models almost perfectly overlapping the reported data. Specifically, at 30 eV, models 3 and 4 show the best agreement with the available results.

While previous studies have demonstrated a good agreement between theoretical calculations and experimental measurements in elastic electron scattering by furan, our results, particularly from the more robust 197-channel model, show enhanced accuracy in specific aspects. The improvements stems from a refined treatment of polarization and multichannel coupling effects, which leads to closer alignment with experimental data. Although the characterization of resonances was not the primary focus of this work, our calculations still yield results that align well with established theoretical and experimental findings. Notably, the 197-channel model predicts two π^*^ shape resonances at 1.96 and 3.69 eV, corresponding to the *B*_1_ and *A*_2_ symmetries, respectively, which are in good agreement with previous results.^29,30,49^

Present results involving the excitation for the two lowest-lying excited states of the furan molecule exhibit a behavior similar to the elastic scattering, with excellent consistency between the cross sections obtained according to model 4 and the measurements reported by da Costa et al.^31^ and by Regeta and Allan^33^ for energies above 7 eV. For energies close to the thresholds of the first two triplet excited states, the calculations exhibit cross sections with magnitudes greater than the experimental results. This energy region is highly sensitive to polarization effects and also to the presence of pronounced structures due to upcoming thresholds, which significantly affect the obtained cross sections. Even considering these factors, the most robust model (namely, model 4 including up to 197 channels) displays excellent agreement with the experimental data reported in the literature. This is particularly true even for energies where a considerable number of electronic states become energetically accessible, an outcome not previously achieved for this system.

Our study highlights the critical role of multichannel coupling and the careful selection of excited states in low-energy electron scattering calculations for furan. While we have not yet reached the computational limits of our code in terms of the number of channels, the key insight lies in the inclusion of states that significantly improve the description of specific target states. The comparison of cross sections across the four multichannel coupling models demonstrates that differences in the strategy for selecting excited states within the FSCI approximation, as well as the number of channels included, can profoundly influence the results. These findings underscore the importance of balancing computational feasibility with the accurate representation of excited states to achieve better agreement with experimental data.

## Supplementary Material



## Data Availability

The data supporting this article are available in the Zenodo repository under the DOI 10.5281/zenodo.14961736. The data set can be accessed at https://zenodo.org/records/14961736.

## Notes

The excitation energies of the furan molecule and computational details (number of FSCI excited states, hole-particle pairs, channels, CSFs per symmetry, and excited state energy ranges) for the scattering calculations performed according to the different models considered in the present work (ZIP)
