# Supplementary material for: Improving the Description of Electronically Inelastic Scattering of Electrons by the Furan Molecule
Source: ACS Phys Chem Au. 2025 Jul 29;5(5):490–500. doi: 10.1021/acsphyschemau.5c00027 (PMC12464759; doi:10.1021/acsphyschemau.5c00027)
Supplement: Supplementary file 1 [file pg5c00027_si_001.zip › Supporting_Information_for_Publication.pdf]

Supporting Information for Publication: Improving the Description of Electronically Inelastic Scattering of Electrons by the Furan Molecule

Yan A. C. de Avó,[*,†] Giseli M. Moreira,[‡,¶] and Romarly. F. da Costa[*,†]

†*Centro de Ciências Naturais e Humanas, Universidade Federal do ABC, 09210-580 Santo André, São Paulo, Brazil*

‡*Departamento de Física, Universidade Estadual do Centro-Oeste, 85040-167 Guarapuava, Paraná, Brazil*

¶*Departamento de Física, Universidade Federal do Paraná, Caixa Postal 19044, 81531-980 Curitiba, Paraná, Brazil*

E-mail: alexssander.yan@aluno.ufabc.edu.br; romarly.costa@ufabc.edu.br,roma.ifi@unicamp.br

List of Tables

S1 Comparison of excited state energies (eV) for furan obtained from different theoretical methods and experimental results. The FSCI results represent our benchmark calculations using the full single-configuration space, contrasted with MOBSCI approximations under different models. 3

S2 Number of FSCI excited states, hole-particle pairs, channels, CSFs per symmetry, and excited state energy ranges for the different models. 3

List of Figures

S1 Density of electronic excited states of furan, calculated using different models for the inclusion of multichannel coupling effects: 47 channels (green), 103 channels (orange), 109 channels (magenta), and 197 channels (cyan). The horizontal bars indicate the thresholds of the excited states within each model. 4

Table S1: Comparison of excited state energies (eV) for furan obtained from different theoretical methods and experimental results. The FSCI results represent our benchmark calculations using the full single-configuration space, contrasted with MOBSCI approximations under different models.

States	MOBSCI				FSCI	MOBSCI [1]	TDDFT [2]	CASPT2 [3]	SAC-CI [4]	SAC-CI [5]	CASSCF [6]	MRD-CI [7]	Experimental		
	47ch	103ch	109ch	197ch									[7]	[8]	[9]
1^3B_2	3.51	3.47	3.48	3.48	3.25	3.68	3.77	3.99	4.40	4.39	–	3.93	3.99	3.97	4.00
1^3A_1	5.04	4.98	5.00	5.00	4.78	5.12	4.99	5.15	5.75	5.63	–	5.28	5.15	5.15	5.20
1^3A_2	6.17	6.13	6.17	6.17	6.08	–	5.69	5.86	6.20	–	–	–	–	–	–
1^1A_2	6.30	6.27	6.30	6.30	6.25	–	5.81	5.92	–	–	5.62	–	–	–	–
1^1B_2	6.92	6.88	6.92	6.89	6.46	7.72	6.00	6.04	–	6.40	7.74	6.76	6.04	–	–
1^3B_1	6.79	6.75	6.79	6.76	6.70	–	6.20	6.42	6.68	–	–	–	–	–	–
1^1B_1	7.15	7.14	7.15	7.14	6.86	–	6.30	6.46	–	–	6.10	–	–	–	–
2^3A_2	7.11	7.09	7.11	7.09	7.05	–	–	–	6.95	–	–	–	–	–	–
2^3A_1	7.47	7.41	7.41	7.41	7.08	–	–	–	7.42	–	–	6.65	–	–	–
2^1A_2	7.25	7.16	7.22	7.17	7.12	–	–	6.59	–	–	6.11	–	–	–	–
2^3B_1	7.61	7.57	7.61	7.60	7.51	–	–	–	7.57	–	–	–	–	–	–
2^1B_1	7.69	7.66	7.69	7.68	7.63	–	–	7.15	–	–	–	–	–	–	–
3^3A_2	7.91	7.79	7.90	7.87	7.71	–	–	–	8.29	–	–	–	–	–	–
2^3B_2	7.97	7.94	7.97	7.97	7.89	–	–	–	6.87	–	–	–	–	–	–
2^1A_1	8.17	8.08	8.16	8.12	8.01	8.39	6.25	6.16	–	6.79	–	6.02	5.80	–	–
⋮	⋮	⋮	⋮	⋮											
Last State	16.71 ($^1B_2$)	19.56 ($^1A_2$)	19.80 ($^1A_2$)	19.90 ($^1B_2$)	–	–	–	–	–	–	–	–	–	–	–

Table S2: Number of FSCI excited states, hole-particle pairs, channels, CSFs per symmetry, and excited state energy ranges for the different models.

Calculations	FSCI States	Hole-Pairs	Channels	CSFs				Excited State Energies (eV)			
				A_1	A_2	B_1	B_2	Triplet		Singlet	
								Lowest	Highest	Lowest	Highest
Model 1	22	23	47	978	1063	995	1068	3.505	15.920	6.301	16.710
Model 2	22	51	103	2047	2366	2083	2396	3.471	19.421	6.270	19.557
Model 3	40	54	109	2747	1928	2785	1945	3.476	19.449	6.300	19.801
Model 4	80	98	197	4728	3742	4724	3735	3.475	19.690	6.297	19.895

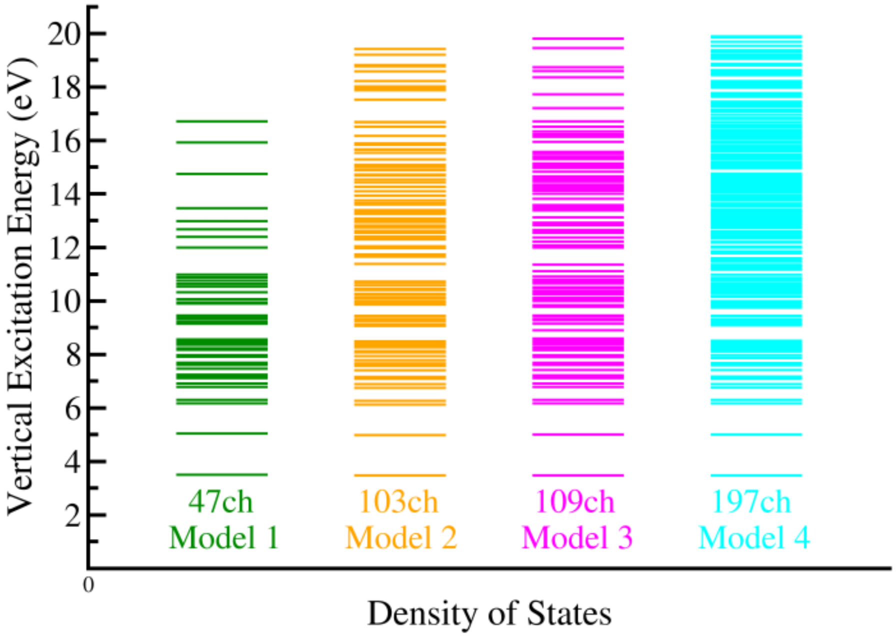

Figure S1: Density of electronic excited states of furan, calculated using different models for the inclusion of multichannel coupling effects: 47 channels (green), 103 channels (orange), 109 channels (magenta), and 197 channels (cyan). The horizontal bars indicate the thresholds of the excited states within each model.

References

(1) R. F. da Costa, M. H. F. Bettega, M. A. P. Lima, M. C. A. Lopes, L. R. Hargreaves, G. Serna and M. A. Khakoo, *Phys. Rev. A*, 2012, **85**, 062706.

(2) N. Gavrilov, S. Salzmann and C. M. Marian, *Chem. Phys.*, 2008, **349**, 269.

(3) L. Serrano-Andres, M. Merchan, I. Nebot-Gil, B. O. Roos and M. Fulscher, *J. Am. Chem. Soc.*, 1993, **115**, 6184.

(4) H. Nakatsuji, O. Kitao and T. Yonezawa, *J. Chem. Phys.*, 1985, **83**, 723.

(5) J. Wan, M. Hada, M. Ehara and H. Nakatsuji, *J. Chem. Phys.*, 2001, **114**, 842.

(6) H. Nakano, T. Tsuneda, T. Hashimoto and K. Hirao, *J. Chem. Phys.*, 1996, **104**, 2312.

(7) M. H. Palmer, I. C. Walker, C. C. Ballard and M. F. Guest, *Chem. Phys.*, 1995, **192**, 111.

(8) W. M. Flicker, O. A. Mosher and A. Kuppermann, *J. Chem. Phys.*, 1976, **64**, 1315.

(9) A. Giuliani and M.-J. Hubin-Franskin, *Int. J. Mass Spectrom.*, 2001, **205**, 163.

(10) E. H. Van Veen, *Chem. Phys. Lett.*, 1976, **41**, 535.
